# Pyrolysis of Linear Copolymers of Ethylene and Propylene^1^

**DOI:** 10.6028/jres.065A.026

**Published:** 1961-06-01

**Authors:** Sidney Straus, Leo A. Wall

## Abstract

The rates of volatilization of linear polymers of ethylene and propylene and their copolymers are somewhat characteristic of random degradation in that a maximum occurs in the rate curve for all polymers studied. Increasing amounts of propylene in the copolymer showed increases in the maximum rate on thermal decomposition. Minute inorganic and organic impurities added to the polypropylene have considerable effect on the thermal stability of the polymer by lowering the rates of volatilization and increasing the activation energy. Possibly there is an inhibition of the free-radical chain process, and the overall reaction becomes more like that for the initiation process. Rate studies and volatile decomposition products for a fully deuterated linear polyethylene were also determined, and its activation energy was calculated to be 70 kcal/mole, which is very close to the value (72 kcal/mole) calculated for the nondeuterated polymer. The effect of a large dose of beta-radiation on a linear polyethylene was also determined. Results suggest that scissions in the chain, as well as cross links, are caused by the radiation.

## 1. Introduction

Although the overall decomposition of linear polyethylene exhibits features indicating random degradation, the decomposition of branched polyethylene shows an entirely different pattern of behavior [1].^2^ Theoretical treatment [2] of the degradation of branched chains, assuming various ratios for the probabilities of rupture of the bonds at the roots and at points distant from the roots of the branches, did not predict the experimentally observed behavior. More recently the same differences were observed between the results obtained from the decomposition of linear polypropylene and of branched polypropylene [3]. The experimental evidence suggests an increase of intramolecular transfer over intermolecular transfer in the decomposition of the branched polymers. A series of polyethylenes with different numbers and lengths of branches were studied earlier [3].

In the present work a linear polyethylene, a linear polypropylene, and four copolymers of ethylene and propylene combined in different percentages were thermally decomposed in order to study the kinetics of the various reactions involved and also to determine the effect on stability of systematically adding methyl side groups to a linear chain. The effects on thermal decomposition were also determined wherein inorganic impurities had been added to the polymers. In addition, thermal studies were made on a fully deuterated polyethylene polymer and on irradiated linear polyethylene.

## 2. Experimental Procedures

The experimental polymers used in this work are listed in table 1. The first six polymers were obtained from the B. F. Goodrich Co. The last two were prepared in our own laboratory. Available data on weight percent of propylene in the copolymers and on approximate molecular weight are indicated. They were all checked for ash content. Another linear polypropylene was also studied which had an appreciable ash content. In this paper it will be referred to as polypropylene-B.

The polytetradeuteroethylene was prepared by polymerizing tetradeuteroethylene in sealed thick-walled hard-glass tubes at 20 °C by exposure to gamma rays for several days. The material received a total dose of approximately 0.5 Mr (megaroentgen). Polyethylenes produced under these conditions show no methyl groups by infrared analysis. However, the polytetradeuteroethylene used in this work was somewhat cross linked.

Linear polyethylene used in previous work (1), which was prepared from diazomethane using boron trifluoride etherate as a catalyst, was irradiated and then pyrolyzed. It was irradiated with 800 kv electrons to a total dose of 30 Mr under nitrogen at the General Electric Co., through the courtesy of Dr. E. J. Lawton.

All the polymers were pyrolyzed in an apparatus wherein loss of sample in weight per minute could be determined by means of a tungsten spring balance inside a heated furnace [4]. In addition, several of the polymers studied were also decomposed in a tube furnace [5], after which the volatile products were analyzed by mass spectrometry.

## 3. Results

**Figure 1:**
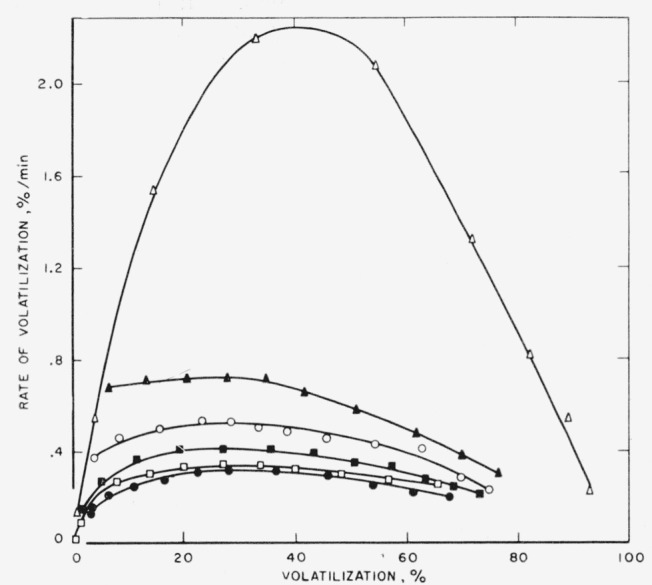
Pyrolysis of copolymers of ethylene and propylene at 400 °C Percentage of propylene in copolymers: ●, 0%; □, 5.9%; ■, 10.9%; ○, 21.3%; ▲, 33.9%; Δ, 100%.

Rates of volatilization were studied on the ethylenepropylene polymers by means of the tungsten spring balance apparatus at 400 °C; the results are shown in figure 1. All the polymers exhibited degradation curves with maxima, which are characteristic of linear olefinic polymers [1, 3]. With increasing amounts of propylene in the polymer the maximum rate of volatilization is increased.

**Figure 2:**
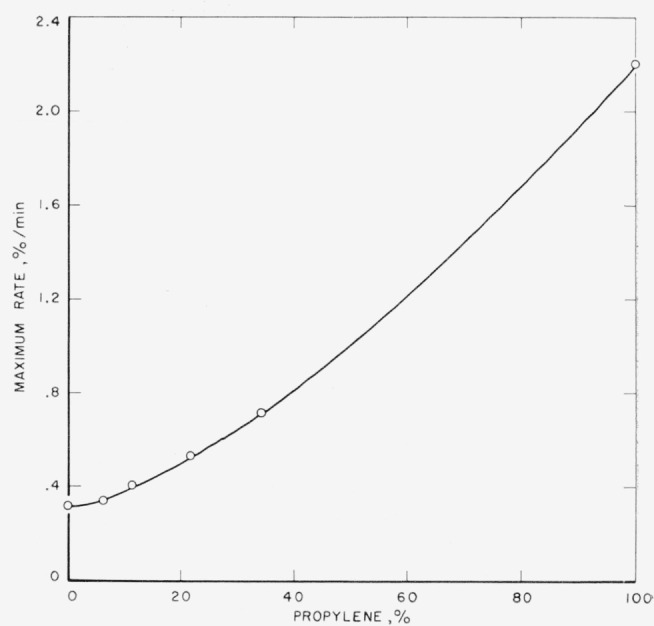
Maximum rates of decomposition at 400 °C versus percent of propylene in copolymers of ethylene and propylene.

Figure 2 shows the maximum rates plotted as a function of the percentage of propylene in the polymers in order to ascertain the relationship between the rates and composition. The downward curvature indicates relatively slower rates for the copolymers than what would occur on an additive basis. This curve may be of some utility for approximating the rates of volatilization at this temperature for other copolymers of ethylene and propylene.

**Figure 3:**
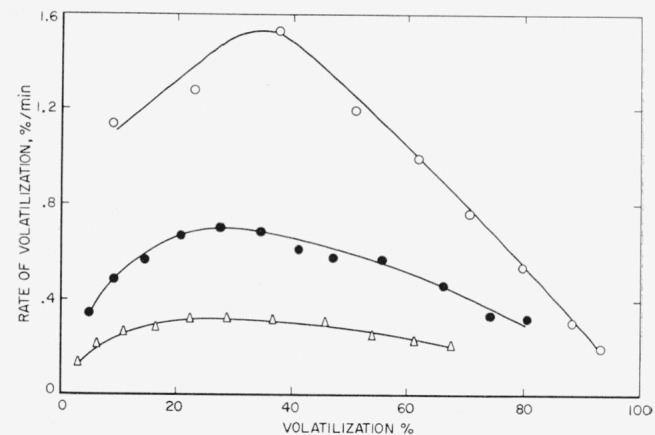
Pyrolysis of linear polyethylene Δ, 400 °C; ●, 410 °C; ○, 420 °C.

**Figure 4:**
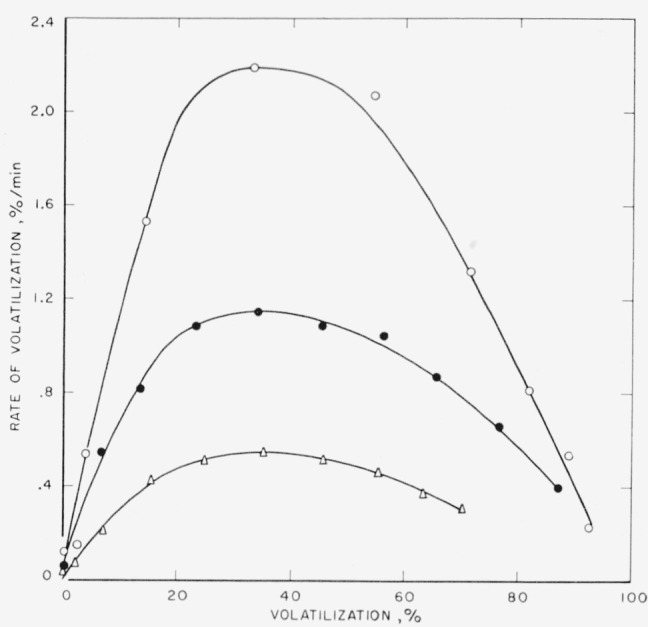
Pyrolysis of linear polypropylene Δ, 380 °C; ●, 390 °C; ○, 400 °C.

Additional rate studies were made on the linear polyethylene and polypropylene polymers. The results are shown in figures 3 and 4. With increase of temperature, the maximum peak heights shift in position from about 25 percent volatilization at 400 °C to almost 35 percent at 420 °C in the case of polyethylene. Figure 3 indicates that some of the polymer is lost prior to the initial reading. This loss may arise from the fact that some of the polymer is degraded during the 15-min heating-up period that is required in order that the sample reach the temperature of pyrolysis [4], or from the presence of a small amount of low molecular weight material or some impurities in the polymer. Using the maximum rates at the three temperatures shown, an activation energy of 72 kcal/mole of linear polyethylene was calculated by means of the Arrhenius equation. This is the same value that was obtained by Madorsky on another linear polyethylene, after correction for temperature [6].

The maximum peak heights for the polypropylene polymer, as can be seen in figure 4, cover a broad area at approximately 30 to 40 percent volatilization. There is only a negligible loss of polymer during the heating-up period in this case, indicating a more clean-cut molecular weight distribution. An activation energy for polypropylene, likewise calculated on the basis of maximum rates at the three temperatures shown, was determined to be 59 kcal/mole, which compares with the value of 58 kcal/mole determined by Madorsky on another polypropylene polymer [6].

Another experimental polymer, designated here as polypropylene-B, was also studied for both rates of volatilization and analysis of its volatile products. The rates were found to be very inconsistent, depending upon the particular piece of sample selected. At similar temperatures the maximum peak heights varied considerably. In searching for the cause of this variability it was ascertained that the polymer contained some inorganic matter that was probably the cause for these inconsistencies. An “ash” analysis of the polymer showed from 1 to 2 percent of inorganic gray material. A spectrochemical analysis of this substance revealed the presence of relatively large amounts of aluminum, intermediate amounts of iron and silicon, and smaller amounts of titanium, magnesium, copper, calcium, tin, and zinc, in approximately that order. Apparently, an aluminum silicate catalyst or other catalysts had not been completely removed from the polymer in the polymerization process. In addition, the inorganic material was not uniformly mixed in the polymer, thus accounting for the conflicting results. Generally, the greater the ash content, the more stable was the particular sample.

It was then decided to observe the effect of the presence of some of this inorganic material on the thermal decomposition of a relatively clean polypropylene polymer. The polypropylene previously studied was selected for this purpose. A very small amount of this inorganic material in the oxide form, approximately 0.2 mg, was allowed to remain at the bottom of the small platinum tray [4] after flaming to drive off the organic portion of the polypropylene-B. Approximately 5 mg of the uncontaminated polypropylene was then placed in the platinum tray and, as before, pyrolyzed at 400 °C. The polymer, in the form of a powder, melts at about 130 °C and flows to become a colorless glass. The rate of volatilization of the polypropylene was definitely lowered when in contact with the inorganic matter. The maximum rate of volatilization was reduced from 2.2 to less than 1 percent/min. In addition, the residue showed a definite brownish discoloration, whereas the pure polymer degraded to give a residue that remained colorless. Apparently a different depolymerization process was taking place while in contact with the inorganic material, and the pyrolysis of the original polymer was being inhibited.

**Figure 5:**
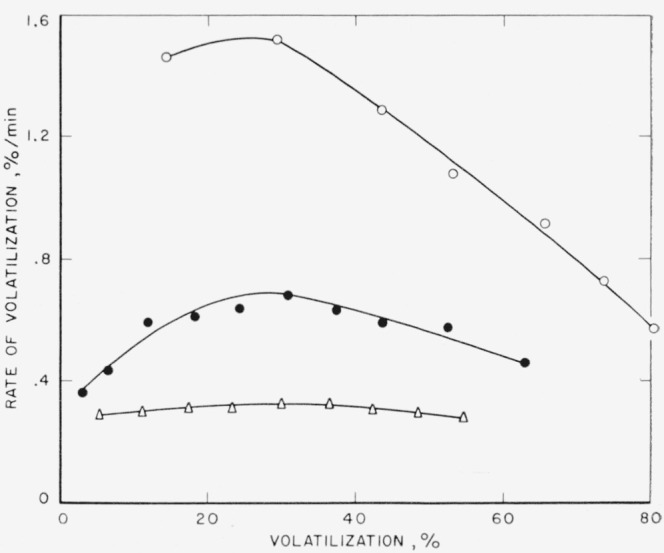
Pyrolysis of polypropylene treated with hot xylene and in contact with inorganic impurities Δ, 390 °C; ●, 400 °C; ○, 410 °C.

Furthermore, it was also determined that after dissolving the pure linear polypropylene in hot xylene, evaporating off the solvent at 110 °C in a vacuum, placing the polymer in contact with the same inorganic material, and then pyrolyzing it at 400 °C the maximum peak height was reduced to approximately 0.70 percent/min. Rates of volatilization of the similarly treated polymer at temperatures of 390, 400, and 410 °C gave an activation energy of approximately 68 kcal/mole. The rates were reduced by a factor of approximately 3 and the activation energy increased by approximately 9 kcal/mole. These results can be seen in figure 5.

**Figure 6:**
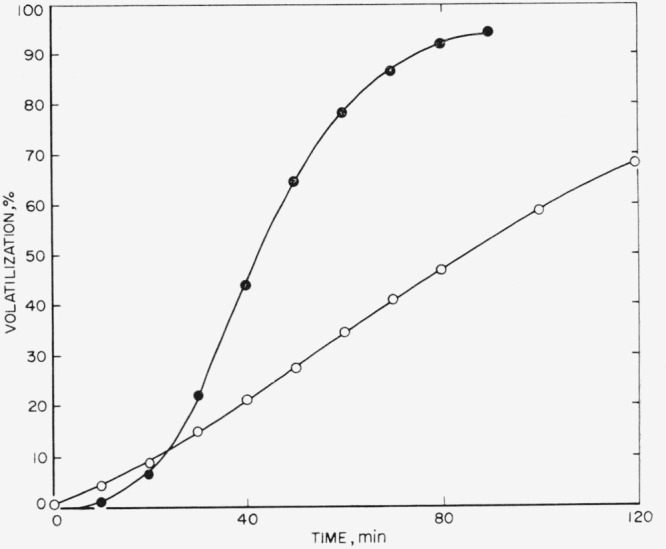
Effect of impurities on amount volatilized versus time for polypropylene at 400 °C ●, Original polymer; ○, treated with hot xylene and in contact with impurities

Figure 6 shows a comparison between the original polymer and the treated polymer when the percent volatilized is plotted as a function of time for 400 °C pyrolysis in the tungsten-spring balance. The original polypropylene has a slower rate of volatilization initially; however, it then becomes rapid; over 90 percent of the polymer is degraded after 90 min. The treated polymer, dissolved in hot xylene, dried, and then pyrolyzed while in contact with the inorganic impurities degrades slightly faster initially but then progresses slower than the original polymer, until after 120 min of pyrolysis only 68 percent of the polymer has been degraded. The kinetics of this mechanism are not clear as yet, but apparently the original free-radical mechanism has been inhibited in some manner so that depolymerization under these new conditions has proceeded in a new direction.

The effect of xylene treatment alone on the rate of degradation of the polypropylene polymer is puzzling. When the polymer is dissolved in xylene and dried, it also yields a lower rate of volatilization at 400 °C, approximately 1.3 percent/min, except that initially there is a greater loss. In addition, the maximum peak heights cover a much broader area than in the case of pyrolysis of the untreated polypropylene. The xylene inclusion into the polymer might inhibit its breakdown if free radicals in the polypropylene react by removing a hydrogen atom from one of the methyl groups of the xylene. This would tend to stabilize the polymer by reducing its later transfer ability on thermal decomposition. The amount of xylene left in the polymer after drying is small, less than 0.1 percent by actual weight, but this evidently is sufficient to reduce the overall rate. The small amount of xylene present may also be the reason for the initial higher rate as compared to the case with no solvent in the polymer. It might be noted here that the presence of inorganic impurity in the linear polyethylene polymer or the treatment of this polymer in hot toluene has very little effect, if any, on its rate of decomposition.

As for the effect of the inorganic impurities, much more work will have to be done in this area of inhibition to polymers in general and to polypropylene in particular. Moreover, individual effects of inorganic materials as well as combined effects will have to be studied. In any event, the observed inhibition effect is most encouraging for future possibilities of stabilization, but theoretical interpretations will have to wait for more sophisticated kinetic experiments.

Polypropylene-B was also pyrolyzed in the tube furnace at 400 °C for a determination of the volatile products. The mass spectra of the light volatiles, about 13 percent of the total volatiles, was somewhat similar to that of a previous analysis obtained on a nonlinear polypropylene [7]. There were carbon compounds from C_1_ through C_9_, some of the larger components being pentane, hexene, pentene, butane, propylene, and ethylene, in diminishing order. The volatiles obtained from the clean polypropylene at 400 °C were also quite similar, except that the yield of monomer was somewhat greater than in the other two cases.

**Figure 7:**
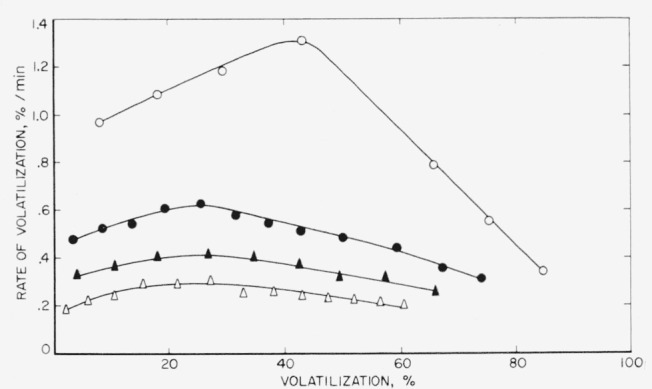
Pyrolysis of polytetradeuteroethylene Δ, 400 °C; ▲, 405 °C; ●, 410 °C; ○, 420 °C.

A study was also made on a fully deuterated linear polyethylene, both for the rate of decomposition and for a mass spectral analysis of its more volatile degradation products. Table 1 gives pertinent facts about the polymer. The rates of volatilization at 400, 405, 410, and 420 °C, shown in figure 7, all show maximum, peaks similar to those for the undeuterated polyethylene (see fig. 3). The maximum peak heights are just slightly lower than those obtained for polyethylene at similar temperatures. The activation energy obtained for the deuterated polymer, based on these maxima, was calculated to be 70 kcal/mole.

This deuterated polyethylene was also pyrolyzed at 375, 425, 450, and 500 °C in the tube furnace and various fractions of the degradation products were collected. The monomer fraction, whose components are volatile at room temperature, amounted to about 3.5 percent of the total volatiles. Mass spectral analysis indicated deuterated compounds with up to 12 carbon atoms. No quantitative analysis could be made because of a lack of patterns for deuterated compounds. However, there was a rough similarity between the spectra of the deuterated polymer and those of the hydrogen polymer. In that case, the heavier deuterated volatiles, the 97 percent waxlike fraction, should have had approximately the same average molecular weight as that previously determined cryoscopically for the hydrogen polymer volatiles, namely about 700 (8).

**Figure 8:**
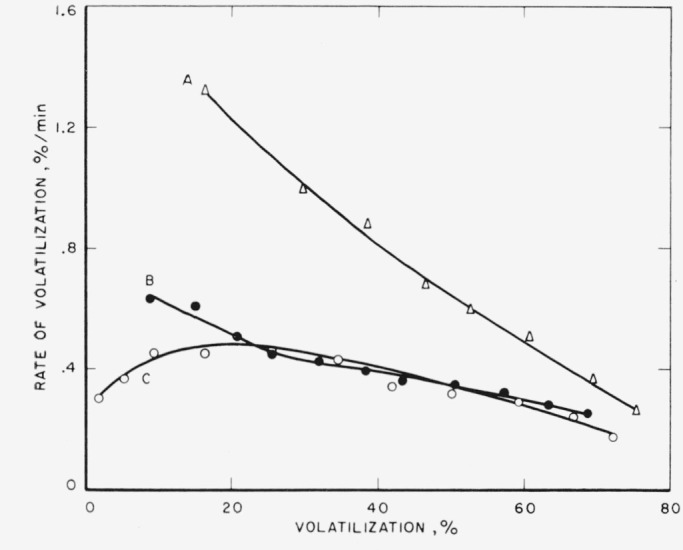
Pyrolysis of beta-irradiated polyethylenes at 400° C ○, Linear unirradiated; ●, linear irradiated; Δ, branched unirradiated and irradiated.

Finally, a linear polyethylene, approximately 1,000,000 molecular weight (see table 1) was given a large dose of radiation and then thermally decomposed at 400 °C in the tungsten-spring balance apparatus. A comparison of the rates with a nonirradiated polymer is shown in figure 8. The irradiated curve no longer shows a maximum as it does in the untreated polymer. Instead, the slope curves downward until about 35 percent volatilization and then almost coincides with the original polymer.

## 4. Discussion

The rates of volatilization, in percent of original sample per minute, for the linear polymers of ethylene and propylene and their copolymers, when plotted as a function of conversion to volatile products, produced curves characteristic, or nearly characteristic, of a net random degradation of linear chains [1,9]. It has been shown [3] recently that branched polyethylene and polypropylene do not exhibit the maximum-type curve but rather a continuously decreasing one, like curve A in figure 8.

Where maxima occur in the rate curves, they are used for evaluating the data because for random degradation a simple relation exists between the rate at the maximum and the overall rate constant for scission, *k.*

$${\left(\frac{dC}{dt}\right)}_{\max}=\frac{kL}{e}.$$

The quantity *L* is the critical size for evaporation [10], i.e., species having a degree of polymerization equal to or greater than *L* must decompose before evaporating, and *e* is the base of the natural logarithms. The value of *L* for polyethylene has been determined to be 72, based on experimental measurement of the products of pyrolysis [10]. For polytetradeuteroethylene *L* is assumed to be same as for polyethylene. For polypropylene, where the monomer unit is larger, the molecular weight of the critical size for evaporation is assumed to be the same as for polyethylene, and therefore its *L* should be 48. A lower thermal stability will decrease *L*, while a lower cohesive energy density will increase it. Whatever assumption one makes as to the value of *L*, provided it is a reasonable one, the error in the calculated rate constant is unlikely to exceed 50 percent.

In table 2 the activation energies, *E*, and pre-exponential factors, *A*, for the overall rate constants are listed for the homopolyers studied. The copolymers of ethylene and propylene were compared only at 400 °C. Because of the lower activation energy for polypropylene it is clear that the difference in rates for the homopolymers of ethylene and propylene will be greater at lower temperatures. The trend in the copolymer rates (fig. 2) shows only a small tendency towards relatively increased stability for the copolymers; i.e., relative to a straight line between the rates for the homopolymers. This may be the result of the elimination of a small steric repulsion between methyl group of the propylene units when they are isolated from one another, as in the copolymers. The activation energies for polyethylene and polypropylene are large enough so that C—C bond dissociation may be occurring as the initiating step in the thermal decomposition. On the basis of a chain reaction for thermal decomposition one can roughly estimate [11] the dissociation energies as $\left(99-2\;E_{2}^{1}\right)$ for polyethylene and $\left(85-2\;E_{2}^{1}\right)$ for polypropylene, where $E_{2}^{1}$ is the activation energy for propagation in free-radical polymerization and is likely to be around 5 kcal/mole.

### 4.1. Irradiated Polyethylenes

Previously it has been observed (1) that branched polyethylenes, when pyrolyzed, produced rate-versus-conversion behavior of the type shown in figure 8, curve *A*, while linear polyethylene produced that in curve *C.* Irradiation of the branched polyethylene to a total dose of 30 Mr showed no change in the rate of volatilization. However, it is now seen that, upon irradiation to 30 Mr, the linear polyethylene degrades to give curve *B*, which resembles *A.* The rates in the initial stages of pyrolysis are increased to an extent that the maxima in the curves are eliminated.

A priori, it is clear that cross linking alone would not lead to this behavior. Therefore some scission is indicated. It is possible that some very small molecules of the order of 1,000 to 5,000 molecular weight may contribute to the observed behavior; however, it is believed that scission combined with cross linking or primary radicals coupling with secondary ones produce a branched structure with long branches. The results support the speculation that long-chain branches are more effective in producing the behavior of the A-type curve than short-chain branching. The irradiation is not likely to produce short chain branches, and the amount of irradiation is unlikely to have produced many branches.

### 4.2. Polytetradeuteroethylene

The polymer polytetradeuteroethylene was produced by gamma-ray-initiated polymerization and was somewhat cross linked. The relatively high rates (see fig. 7) during the early stages of pyrolysis may be the result of the phenomena seen with the irradiated linear polyethylene (fig. 8). It was not expected that deuteration would produce a relatively large detectable effect in this polymer as it did in polystyrene [12]. Isotope effects on the elementary processes may cancel out and not affect the overall rate. For instance, the expected reduction in the rate of transfer may have been compensated by an increase in the rate of initiation. Evidence for an increase in the dissociation rate of hexadeuteroethane into trideuteromethyl radicals over that of ethane has been reported [13]. In polystyrene the monomer yield on degradation increases from 40 to 70 percent upon alpha deuteration [14]. Since the monomer yield for linear polyethylene is of the order 0.1 percent [15] the yield from polytetradeuteroethylene would not be expected to exceed 0.2 percent. Detection of such a change in the present experiments would be extremely difficult because of minor variations in the pyrolytic technique. Lack of mass spectra for larger deuterocarbons which interfere with the mass spectrometric analysis of tetradeuteroethylene also hindered monomer yield determination for this polymer. No appreciable difference was observed in the distribution of pyrolysis products between polyethylene and polytetradeuteroethylene.

### 4.3. Inhibition Effect

The inhibition effect of impurities derived from the catalytic agents used in preparing the polypropylene is difficult to understand. The changes produced in the activation energy and pre-exponential factor are compatible with an inhibition of a free-radical chain process, where the overall activation energy becomes closer to that for the initiation process. One suspects that the impurities may have some dehydrogenating effect. The fact that xylene has a small effect in spite of its volatility at the temperature of pyrolysis suggests radical abstraction of hydrogen in the methyl groups of xylene, producing some stable unreactive radicals. Unsaturated groups in the polymer may have a similar effect.

These results contrast those reported [16] for the polyamides, where traces of acid-polymerization catalyst tend to accelerate thermal decomposition. With polytetrafluorethylene some inhibition has been observed [17] when the material is pyrolyzed under fluorine or hydrogen.

## Figures and Tables

**Table 1:** Polymers investigated

Polymer	Propylene content	Approx. mol wt

	*wt %*	
Polyethylene^a^	0.0	50,000
Polyethylene-propylene^a^	5.9	100,000
Do^a^	10.9	100,000
Do^a^	21.3	100,000
Do^a^	33.9	100,000
Polypropylene^a^	100.0	50,000
Polytetradeuteroethylene^b^	……………….	cross linked
Irradiated linear polyethylene^c^	……………….	cross linked; (10^6^ before irradiation)

a Linear Ziegler-type polymers obtained from the B. F. Goodrich Co.

b Prepared with γ-irradiation.

c Irradiated with 800 kv electrons to a dose of 30 Mr.

**Table 2:** Activation energies for thermal decomposition

Polymer	Rate at 400 °C	L	A	E

	%/*min*		*sec*^−1^	*kcal/mole*
C_2_H_4_	0.32	72	10^17.6^	72
C_2_D_4_	.30	72	10^16.9^	70
C_3_H_6_	2.20	48	10^14.4^	59
C_3_H_6_ (treated)	0.69	48	10^16.8^	68

## Footnotes

2 Figures in brackets indicate the literature references at the end of this paper.
